# From Elemental Sulfur to Hydrogen Sulfide in Agricultural Soils and Plants

**DOI:** 10.3390/molecules24122282

**Published:** 2019-06-19

**Authors:** Laura Olivia Fuentes-Lara, Julia Medrano-Macías, Fabián Pérez-Labrada, Erika Nohemí Rivas-Martínez, Ema Laura García-Enciso, Susana González-Morales, Antonio Juárez-Maldonado, Froylán Rincón-Sánchez, Adalberto Benavides-Mendoza

**Affiliations:** 1 Departamento de Nutrición Animal, Universidad Autónoma Agraria Antonio Narro (UAAAN), Saltillo 25315, Mexico; loflara@gmail.com; 2 Doctorado en Ciencias en Agricultura Protegida, UAAAN, Saltillo 25315, Mexico; jmedmac@gmail.com (J.M.-M.); fabperlab@outlook.com (F.P.-L.); 3 Departamento de Botánica, UAAAN, Saltillo 25315, Mexico; erika_rivas257@outlook.com (E.N.R.-M.); juma841025@gmail.com (A.J.-M.); 4 Arysta LifeScience, Saltillo 25290, Mexico; emlaugaren@gmail.com; 5 CONACYT-UAAAN, Departamento de Horticultura, UAAAN, Saltillo 25315, Mexico; qfb_sgm@hotmail.com; 6 Departamento de Fitomejoramiento, UAAAN, Saltillo 25315, Mexico; frincon@uaaan.edu.mx; 7 Departamento de Horticultura, UAAAN, Saltillo 25315, Mexico

**Keywords:** plant nutrition, sulfate, sulfite, plant health and nutrition, nutraceuticals, polysulfanes, polysulfides, soil microbiome

## Abstract

Sulfur is an essential element in determining the productivity and quality of agricultural products. It is also an element associated with tolerance to biotic and abiotic stress in plants. In agricultural practice, sulfur has broad use in the form of sulfate fertilizers and, to a lesser extent, as sulfite biostimulants. When used in the form of bulk elemental sulfur, or micro- or nano-sulfur, applied both to the soil and to the canopy, the element undergoes a series of changes in its oxidation state, produced by various intermediaries that apparently act as biostimulants and promoters of stress tolerance. The final result is sulfate S^+6^, which is the source of sulfur that all soil organisms assimilate and that plants absorb by their root cells. The changes in the oxidation states of sulfur S^0^ to S^+6^ depend on the action of specific groups of edaphic bacteria. In plant cells, S^+6^ sulfate is reduced to S^−2^ and incorporated into biological molecules. S^−2^ is also absorbed by stomata from H_2_S, COS, and other atmospheric sources. S^−2^ is the precursor of inorganic polysulfides, organic polysulfanes, and H_2_S, the action of which has been described in cell signaling and biostimulation in plants. S^−2^ is also the basis of essential biological molecules in signaling, metabolism, and stress tolerance, such as reactive sulfur species (RSS), SAM, glutathione, and phytochelatins. The present review describes the dynamics of sulfur in soil and plants, considering elemental sulfur as the starting point, and, as a final point, the sulfur accumulated as S^−2^ in biological structures. The factors that modify the behavior of the different components of the sulfur cycle in the soil–plant–atmosphere system, and how these influences the productivity, quality, and stress tolerance of crops, are described. The internal and external factors that influence the cellular production of S^−2^ and polysulfides vs. other S species are also described. The impact of elemental sulfur is compared with that of sulfates, in the context of proper soil management. The conclusion is that the use of elemental sulfur is recommended over that of sulfates, since it is beneficial for the soil microbiome, for productivity and nutritional quality of crops, and also allows the increased tolerance of plants to environmental stresses.

## 1. Introduction

Sulfur is one of the most abundant elements on Earth and is an essential element for living beings, of which constitutes on average 1% of dry weight. In plants, S content varies strongly between species, ranging from 0.1 to 6% of dry weight (0.03 to 2 mmol g^−1^ dry weight) [1]. S belongs to the VIA group of the periodic system, where it is found together with O, Se, Te, and Po; naturally, S is a mixture of four isotopes, ^32^S, ^33^S, ^34^S, and ^35^S. The natural abundance of each is 95.1%, 0.74%, 4.2%, and 0.016%, respectively. Sulfur exists in oxidation states ranging from +6 to −2 (Table 1), with the most oxidized state in the form of sulfate (SO_4_^2−^), which is the chemical form that plants absorb from the soil to feed themselves with S [2].

Biological molecules, which range from small molecules to proteins and other polymers, contain S in its more reduced states 0, −1, and −2. For example, it is known that approximately 40% of enzymes depend for their catalytic activity on the presence of sulfhydryl groups (-SH). These -SH groups participate in redox reactions, provide binding sites for toxic or physiologically important metals, and are related to the detoxification of various xenobiotics. It is also known that the tertiary and quaternary structure of many proteins is the result of the presence of disulfane bonds (-S-S-) formed by the oxidation of -SH groups of cysteine, a sulfur amino acid that, together with methionine, is a key factor in determining the nutritional value of plants, as well as a central element in the metabolism of S in all organisms [2].

For the above reasons, a close relationship between nitrogen and sulfur nutritional status has been found in plants [3,4]. Approximately 80% of nitrogen and sulfur incorporated in organic compounds of plants is found in proteins when both elements are in adequate proportions. The S/N balance of a plant, described by the organic S/N ratio, is in the range of 0.025 (legumes) to 0.032 (grasses) and is relatively constant from one species to another. Therefore, the amount of S required by a plant is strongly dependent on its N nutrition. The consequence is that the availability of S below the needs of the crops does not allow the adequate use of applied N [5].

Compounds as important as β-lactam antibiotics (penicillins, cephalosporins, and cephamycins) have an S atom derived from cysteine. The sulfur compound S-adenosyl-l-methionine (SAM) is the most crucial methylating agent known in all organisms; SAM-mediated transmethylation reactions are essential in the regulation of gene expression, the activity of various enzymes, the synthesis of compounds such as the osmolyte DMSP (dimethyl sulfoniopropionate) and DMS (dimethyl sulfide) gas, as well as in the production of antibiotics [2].

The Earth’s S stores are located in the lithosphere, hydrosphere, atmosphere, and biosphere. Human activities result in the extraction of S from the lithosphere (burning of fossil fuels, mining of elemental S and metals) and biosphere (oxidation of organic matter from the soil and burning of biomass). Anthropogenic S is incorporated into the global cycle mainly in the form of SO_2_ emitted into the atmosphere [6].

**Figure 1:**
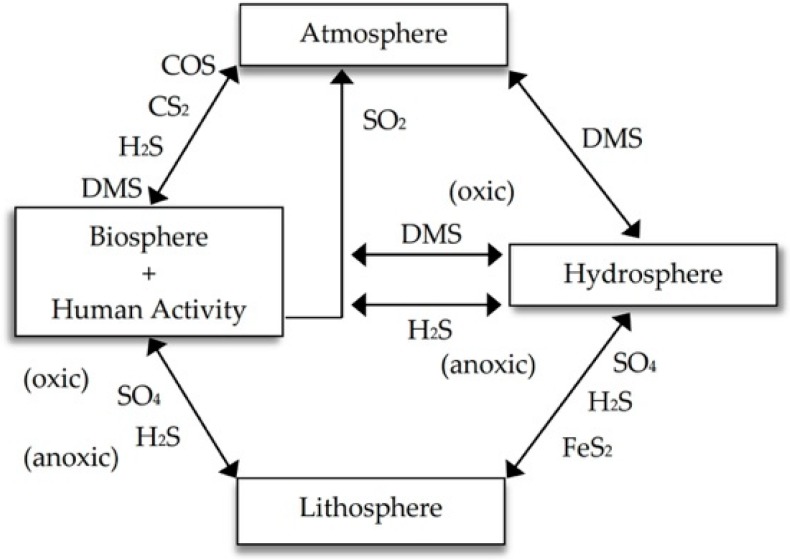
Simplified biogeochemical sulfur cycle. Human activities, fauna, vegetation, and soil microorganisms can be visualized as an interface (as source and sink) to accelerate the transfer of sulfur species between the lithosphere, atmosphere, and hydrosphere.

Between the terrestrial and marine masses, there is a constant flow of S via the atmosphere through the gaseous forms of the element (SO_2_, COS, H_2_S, DMS, and CS_2_) [6] and aerosols (mainly SO_4_^2−^ from the oxidation of sulfur gases, and <10% of organosulfates) [7], or by runoff from terrestrial to oceanic regions (Figure 1). The constant mobilization of S causes changes in the sulfur species that move from one terrestrial compartment to another. Under oxic conditions, the predominant inorganic form of S is SO_4_^2−^, resulting from atmospheric deposition or oxidation of reduced forms of S. In the soil, continuous land tillage that oxidizes soil organic matter and repeated extractions for crops cause the decrease of S stores; for this reason, the regular application of S with the fertilizers is recommended [8].

In soils, most S is found in organic forms; the inorganic forms are elemental sulfur (S^0^) or SO_4_^2−^, the latter can be found as gypsum or be adsorbed in the inorganic exchange matrix. The SO_4_^2−^ adsorbed in the soil is in dynamic equilibrium with the soil solution, and the adsorption/desorption quotient inversely depends on the pH value of the soil and the cations present in the exchange matrix, showing higher affinity for Al^3+^ > Ca^2+^ > K^+^ [9,10].

In soil, SO_4_^2−^ is subject to dissimilatory and assimilatory reduction. Dissimilatory reduction occurs when SO_4_^2−^ is used as a final acceptor of electrons in the anaerobic metabolism of microorganisms, producing H_2_S that is reoxidized in the presence of O_2_ or volatilized into the atmosphere. Assimilatory reduction is used by prokaryotes, algae, plants, and fungi for the biosynthesis of organic compounds, e.g., amino acids. Animals and protists cannot perform assimilatory reduction of SO_4_^2−^; therefore, they depend on the organic sulfur compounds synthesized by other organisms [6]. In many crop species, sulfur is an element associated with nutritional quality and density of mineral nutrients, tolerance to stress, and the management of certain pests and pathogens [11,12,13].

In agricultural soils, SO_4_^2−^ used by crop plants comes mainly from the contribution of fertilizers with sulfates, such as ammonium sulfate, gypsum, potassium sulfate, magnesium sulfate, single superphosphate, ammonium phosphate sulfate, potassium magnesium sulfate, and sulfates of micronutrients [8,14], as well as the oxidation of S^0^, and of S^2−^ contained in organic fertilizers. Another part of the S of crops is obtained from SO_4_^2−^ and aerosols coming from precipitation, as well as the absorption by soil and plants of aerosols and gases such as H_2_S, COS, and DMS. When S is added to the soil in the form of SO_4_^2−^, plants and aerobic prokaryotes absorb it and incorporate it into a reductive metabolism that produces sulfide (S^2−^). On the other hand, when S is supplied as S^0^ or in the form of organic fertilizers (S^2−^), it must be oxidized to SO_4_^2−^ by the action of soil prokaryotes to be available to plants [2,6,8].

The aim of the present review is to describe the dynamics of sulfur in soil and plants, considering elemental sulfur as the starting point and, as a final point, the sulfur accumulated as S^−2^ in biomolecules and biological structures, transformed into myriad sulfur compounds and returned to atmosphere and hydrosphere as H_2_S and other gaseous molecules. The factors that modify the behavior of the different components of the sulfur flow in the soil–plant–atmosphere system are described, along with how these influences the productivity, quality and stress tolerance of crops.

## 2. Transformations of Elemental Sulfur in Soil

**Figure 2:**
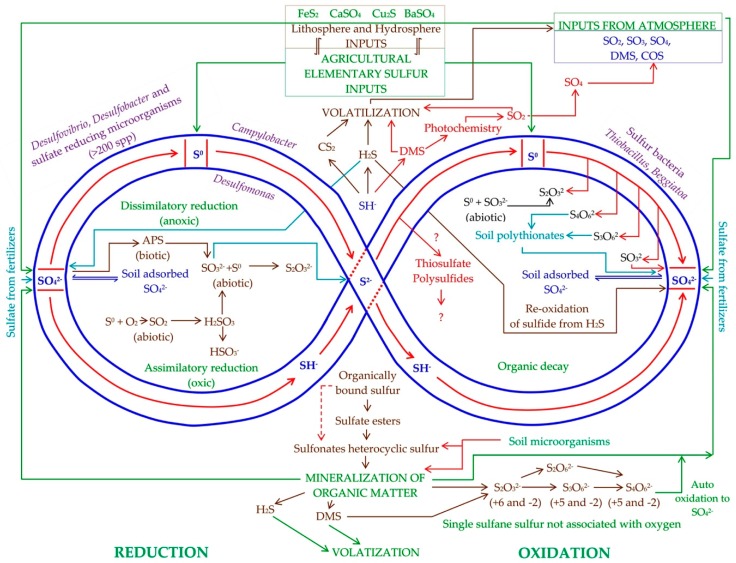
Schematic representation of the flow of sulfur in soil. APS = adenosine 5′-phosphosulfate. Oxidation states of sulfur in the different molecules are: SO_4_^2−^ (+6); S_2_O_6_^2−^ (+5 and −2); S_4_O_6_^2−^ (+5 and −2); S_3_O_6_^2−^ (+5 and −2); SO_3_^2−^ (+4); SO_2_ (+4); S_2_O_3_^2−^ (+6 and −2); COS (+2); S^0^ (0); SH^−^ (−2); S^2−^ (−2); DMS (−2); CS_2_ (−2).

The S available for plants in agricultural ecosystems is in dynamic storage (Figure 2). It comes from gaseous forms and aerosols of S from the atmosphere, from dissolved S (mostly SO_4_^2−^) in rain and snow precipitation, and from SO_4_^2−^, which is obtained from the oxidation of S of soil organic matter and S^0^. Sulfates can be fixed in the soil exchange matrix or leached to the subsoil [10]. In arid regions, SO_4_^2−^ can be stored in large quantities as gypsum in the subsoil, but in areas with higher water availability, leached SO_4_^2−^ is mobilized to lower horizons and to the subsoil [15].

In the anoxic zones of the soil, S^0^ and SO_4_^2−^ are transformed to H_2_S that is volatilized or is reoxidized to S^0^ y and sulfate in the oxic zone. Plants and microorganisms take the SO_4_^2−^ and reduce it to S^2−^ to incorporate it into a huge variety of organic compounds. Subsequently, these same plants and microorganisms transform a part of the sulfur to H_2_S, DMS, and CS_2_ [8,16]. The above volatile molecules have been associated with detoxification metabolism, stress tolerance, and signaling in plants and prokaryotes [17,18]. As with iodine [19], soil organic matter can transform the S to volatile forms by means of abiotic reactions, but the rate of transformation is very low in comparison with biotic metabolism of S [16].

Since there are several access ways by which S can enter the agricultural ecosystem, it is not possible to mark a specific starting point. Therefore, arbitrarily, the assumption of an application of S^0^ to the soil is taken, and the transformations that this material experiences up to SO_4_^2−^ are described. Once in the form of SO_4_^2−^, it is assimilated into plant cells in the form of myriad organic compounds. The final part of the flow of S from soil to plants ends with the production of volatile compounds by plant cells, or in the transformation of the S contained in plant waste (Figure 2).

S atoms tend to avoid double bonds, therefore, in the S^0^, instead of forming molecules of S_2_ (S=S) the S atoms are grouped in the form of cyclic allotropes (cyclosulfur) or as long chains Sn (catena sulfur) [20]. The S^0^ used to apply to soil consists mainly of molecules of S_8_ (cycloocta-S) that are grouped, forming polymers of variable size; S_8_ is the most stable form from a thermodynamic point of view. S_8_ is a very electrophilic Lewis acid, so it reacts with nucleophilic anions or Lewis bases such as OH^−^, sulfides (S^2−^), thiols (R-SH), thiolates (RS^−^), I^−^, CN^−^, and SO_3_^2−^ [21,22].

S^0^ is applied to the soil or substrate in quantities ranging from 20 to 250 kg ha^−1^ yr^−1^, the last figure being equivalent to 200 mg S^0^ kg^−1^ soil. Once in the soil or substrate, S^0^ begins to transform into other chemical forms, mainly through biotic processes, and, to a lesser extent, by abiotic processes. The transformation rate is inversely proportional to the particle size and directly proportional to the temperature (Q_10_ = 4.0), humidity availability, and abundance of edaphic microorganisms [8,23,24].

Any factor that decreases bacterial activity, such as temperatures <10 °C or >40 °C and lack of humidity in the soil, will reduce the transformation of S. Flooded or compact soils will have anoxic conditions that induce high rates of conversion of S^0^ and SO_4_^2−^ into gaseous forms of sulfur [8,24]. The metabolism of S in soils can modify other processes, as in rice paddies, where the use of gypsum amendment has been shown to decrease greenhouse methane emissions [25]. In alkaline soils, it has been observed that the use of S^0^ induces acidification (by H_2_SO_4_), which increases the bioavailability of elements such as P [26].

When it is desired that S^0^ produces SO_4_^2−^ rapidly available for crops, an S^0^ source with a small particle size (<150 μm or 100 mesh) should be chosen. Contrarily, if a long-term impact (two or more consecutive crops) is sought, it is desirable to use S^0^ sources with a larger particle diameter, or even granular forms such as S^0^ prills or S^0^-fortified N-P-K and DAP fertilizers [27,28]. At a temperature of 14 °C, it was found that, in 51 weeks, 51% of S^0^ with particle diameter 41 μm (300 mesh) was oxidized, compared to 18% of S^0^ with 125 μm (120 mesh). In soils with low temperatures, S^0^ sized 41 μm will oxidize at a rate equivalent to S^0^ sized 125 μm in soils with higher temperatures [23]. In another experiment, applying 50 kg ha^−1^ of S^0^, it was found that 80–90% of S^0^ with particles <150 μm was oxidized over a period of 340 days [29].

On the other hand, it has been found that repeated applications of S^0^ to soil increase the population and the activity of oxidizing bacteria of S^0^ [24]. Accompanying the increase in S^0^ oxidant bacteria was a reduction in the number of fungi and protists, while bacterial and actinomycete populations remained stable [30]. Other authors reported a decrease in biomass and bacterial metabolism by applying S^0^ annually for five years [31].

When S^0^ is in micronized form (<177 μm, <80 mesh) it is used for the control of mites and some fungi [32,33]. The reactivity of micronized S^0^ is a consequence of the high quotient surface/volume of the particles, estimated to be 1300 to 1940 cm^2^ g^−1^ for S^0^ of 125 and 41 μm, respectively [23]. Micronized S^0^ can be applied through the foliar route or even by using pressurized irrigation systems to incorporate it into the soil [34,35]. When applied by irrigation system, the problems associated with the application of micronized S^0^ (because it is a flammable and irritant material) by dusting machines are reduced [24].

The use of S nanoparticles for the control of pathogens in plants has also been described [36,37]. Taking into account the high value of the surface/volume ratio of S nanoparticles, furthermore being a source of S for rapid assimilation by plants and microorganisms, it is possible that they function as biostimulants [38], and that they provide highly reactive S^0^ that works as a tolerance-inducing factor against pathogenic fungi [32,39].

To be available for plants, S^0^ applied to the soil or substrate must be oxidized to SO_4_^2−^. The change in the oxidation state of sulfur from 0 to +6 allows reduction equivalents to be obtained (8H^+^ + 6e^−^). The oxidation is carried out by most soil microorganisms, highlighting *Thiobacillus*, *Beggiatoa*, *Desulfomicrobium*, and *Desulfovibrio*, as well as other heterotrophic aerobics S-oxidizing bacteria such as *Bacillus*, *Pseudomonas*, and *Arthrobacter* [2,40]. Two metabolic pathways have been described that allow the oxidation of inorganic S to SO_4_^2−^: the Kelly–Friedrich pathway, which does not involve the production of intermediates such as polythionates, and the Kelly–Trudinger pathway, which includes as an intermediate output tetrathionate (S_4_O_6_^2−^) and other polythionates [16]. The existence of two different routes and the large number of taxa that carry out the oxidation of S^0^ allow a high redundancy, and capacity to tolerate extensive changes in pH and salinity in soils [41,42].

In Figure 2, the oxidation activity from S^0^ to SO_4_^2−^ is presented on the right side, and shows the Kelly–Trudinger pathway with the production of polythionates such as S_4_O_6_^2−^ and S_3_O_6_^2−^ (as well as S_2_O_3_^2−^ and SO_3_^2−^), which serve as a source of reducing potential and possibly act as inducers of stress tolerance in plants, perhaps by containing a single sulfane sulfur not associated with oxygen [16]. In this regard, Li et al. [43] described polythionates as agents with antibiotic action, the efficacy of which is variable according to the pH. Additionally, the abiotic oxidation of S^2−^ in the presence of S^0^ produces polysulfides [44], which have been described as agents associated with stress tolerance in animal cells [45]. Polysulfides possibly fulfill a similar stress-protection function in plants [46]. It is possible that the presence of S^0^ and S^2−^ in polysulfides [44] explains their ability to induce stress tolerance. The production of polythionates and polysulfides represents an additional advantage of the use of S^0^ as a source of sulfur for crops.

Under anoxic conditions, S^0^ is produced as part of the dissimilatory reduction of SO_4_^2−^. Later, the S^0^ can be assimilated into S^2−^ that will be part of the biomolecules, or it will be volatilized in the case of excess S (see the central section of Figure 2). At the left and right ends of Figure 2, in the central part, the SO_4_^2−^ from fertilizers, precipitation, and mineralization of organic matter is represented. A portion of this SO_4_^2−^ forms a soil adsorbed sulfate storage, which will be in dynamic equilibrium with SO_4_^2−^ dissolved in the soil solution. Under oxic conditions, SO_4_^2−^ is assimilated in S^2−^ by assimilatory reduction and then transformed back into SO_4_^2−^ during the organic decay and mineralization of organic matter [42].

As part of the processes of organic decay, mineralization of organic matter, and sulfate reduction, both the soil, through abiotic reactions, and micro-organisms and plants can be source or sink of volatile forms of S, such as H_2_S, DMS, COS, CS_2_, and SO_2_ (Figure 2). Generally, under anoxic conditions, the oxidized forms of S are reduced by the soil microbiome to H_2_S, CS_2_, COS, DMDS, methyl mercaptan, and COS [8]. These gaseous molecules are believed to be part of a mechanism of dissipation of excess S, although participation in other processes is not ruled out [16,47].

In terms of reductive and oxidative microbial reactions, the most abundant forms of sulfur in the soil and the edaphic microbiome are S^2−^, R-SH, RSSH, polysulfides (RS_n_^2−^), S_2_O_3_^2−^, SO_3_^2−^, SO_4_^2−^ and polythionates [16]. SO_4_^2−^ applied as fertilizer or obtained through the processes described above is the form of S that plants assimilate through their roots [40].

At best growth conditions, a plant’s sulfur requirement ranges from 2 to 10 μmol g^−1^ plant fresh weight day^−1^ [1]. As the flow of S is a dynamic process where the ecosystem receives S from the atmosphere, precipitation, subsoil water, and fertilizers, and loses S through the process of volatilization of S by soil and plants and by leaching, it is difficult to estimate the actual amount of S that a plant surface absorbs, assimilates, leaches, and volatilizes. As an exercise, let us suppose a single sampling point for a field of maize, for example before the harvest. In one hectare, there may be 78,000 kg of fresh weight ha^−1^, which would be equivalent to 25 kg of sulfur contained in the plants. However, the 25 kg ha^−1^ accumulated in the plant tissues at that specific sampling time does not include the S volatilized by the plant itself, or that leached, assimilated, or volatilized in the soil and by the microorganisms.

The point to highlight with the data of the previous paragraph is that the S of the soil is in constant exchange and extraction by the crops, atmosphere, and soil water. Therefore, a continuous supply of S is required, which is recommended to be applied in the form of S^0^ (40–60 kg ha^−1^) every one or two years, to maintain the edaphic store.

## 3. Absorption and Assimilation of Sulfur in Plants

### 3.1. Sulfur Absorption and Transport

The absorption of S from atmospheric sources such as COS, SO_2_, DMS, and H_2_S, can represent a valuable contribution of sulfur for many plants. However, most of the S taken by the plants comes from SO_4_^2−^ dissolved in the soil solution [40,47].

**Figure 3:**
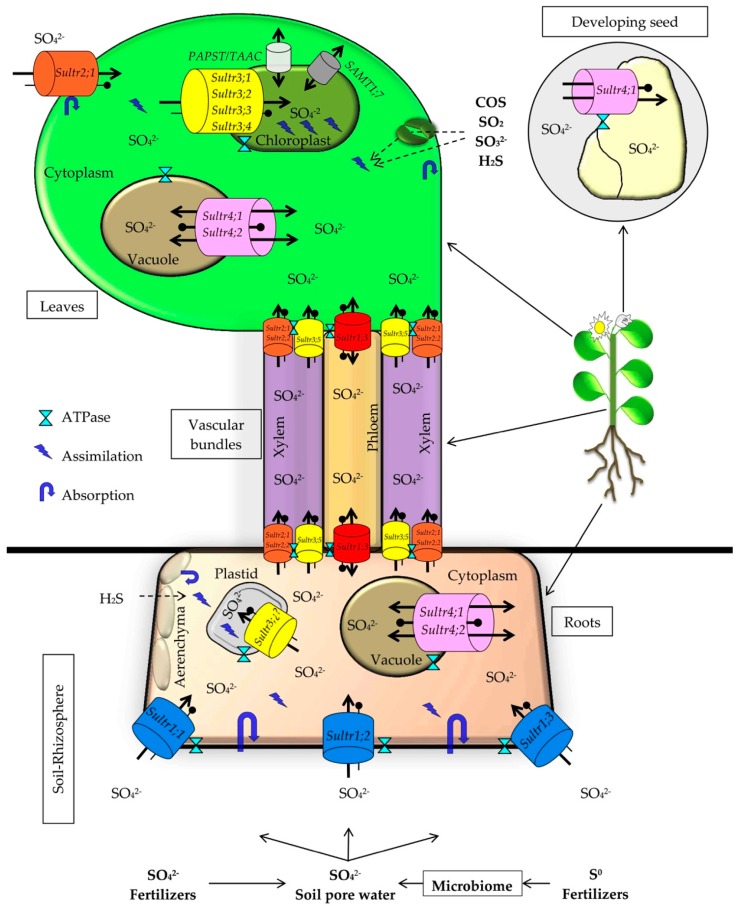
Schematic representation of the processes of absorption, transport, and storage of sulfate.

The SO_4_^2−^ dissolved in the soil solution is absorbed by H^+^/sulfate cotransporters called SULTRs (Figure 3). Plant SULTRs are encoded by a multigene family. SULTRs include high-affinity transport proteins (HAST), low-affinity transport proteins (LAST), vacuole transporters, and plastid membranes and endosymbionts transporters [48,49]. The level of SO_4_^2−^ in the soil solution that induces high-affinity transporters is <10 mg L^−1^ (0.1 mM) [1]. In the soil solution of agricultural areas under strong fertilization management, values of 40–200 mg L^−1^ SO_4_^2−^ are found, while for non-agricultural fertile soils, concentrations of SO_4_^2−^ of 4.5–40.5 mg L^−1^ were reported in the soil solution [50]. The assimilation of S shows a high degree of control and coordination with the assimilation of C and N, and in root transporters there seems to be a relevant regulatory site [1,49].

HAST Sultr1;1, Sultr1;2, and Sultr1;3 facilitate the absorption of SO_4_^2−^ in the root. The HAST of the epidermis and the cortex is accompanied by the LAST Sultr2;1, Sultr2;2, and Sultr3;5, with which they act synergistically. HAST are very abundant in the epidermis and cortex of the root, while LAST proliferate in the parenchyma adjacent to the xylem and phloem [49,51]. The SO_4_^2−^ absorbed is stored in the vacuoles thanks to the co-transporters Sultr4;1 and Sultr4;2 [49,51,52], or it is distributed to the rest of the plant, depending on the sink tissues’ demand. Translocation from the root to the stems and leaves through the xylem occurs through Sultr1;3, Sultr2;1, Sultr2;2, and Sultr3;5 [49,51,53,54,55]. In most species, the absorbed sulfate is assimilated largely into proteins and other biomolecules. However, in others, such as *Brassica oleracea* seedlings, it is possible to observe high amounts of SO_4_^2−^ in the plant tissues [1].

The discharge of SO_4_^2−^ from the xylem to the mesophyll cells of the leaf is mediated by HAST and LAST (Sultr1;3, Sultr2;1, Sultr2;2, and Sultr3;5). As occurs in the root, a part of the sulfate is stored in the vacuoles of stems and leaves by Sultr4;1, and Sultr4;2 [49,51,52], while a part is taken to the chloroplasts by the co-transporters Sultr3;1, Sultr3;2, Sultr3;3, and Sultr3;4 [49,51,55,56], where it will be reduced to S^2−^ to be assimilated into biological molecules [56]. According to the plant’s needs, the sulfate stored in the vacuoles can be re-mobilized through the co-transporters Sultr4;1 and Sultr4;2 [51,52].

The capacity of sulfur absorption, assimilation, and volatilization responds to the nutritional status of the plant. In turn, the sulfur nutritional status of the latter depends on the growth rate and the interaction with the C and N levels of the plants. Biomolecules synthesized from C and N assimilated during photosynthesis are the primary sink for S, and probably constitute part of the signals that regulate SO_4_^2−^ absorption, transport, and assimilation [1]. It is assumed that an excess of S will trigger a higher accumulation of SO_4_^2−^ in the vacuoles, as well as an increase in the synthesis of volatile forms of S, such as H_2_S [16,47]. On the other hand, under S deprivation there will be a significant increase in the expression of sulfate transporters, which will increase the absorption and assimilation of SO_4_^2−^ [1].

Other environmental factors, which possibly modify the absorption of SO_4_^2−^, also regulate the relative abundance of HAST and LAST. Among these factors are salinity, drought, and high temperature in maize [57], drought and salinity in *Arabidopsis* and *Medicago truncatula* [58], and heavy metals such as Cd in sorghum [59].

Considering now the absorption of gas molecules of S, atmospheric SO_2_ can be absorbed through the stomata. In the water film of the substomatal chamber, it is transformed into HSO_3_^−^ and SO_3_^2−^, which is incorporated into the sulfur reduction pathway to be reduced to S^2−^. Another alternative is that SO_3_^2−^ can be oxidized extra- and intracellularly to SO_4_^2−^ by peroxidases, or non-enzymatically by O_2_^−^ radicals or metal ions. This new SO_4_^2−^ is again incorporated into sulfur reduction pathway or transferred into the vacuole. A high level of SO_4_^2−^ is typical in plants exposed to SO_2_ [1,60,61].

Similarly, by diffusion, H_2_S from the soil, vegetation, or atmosphere can be absorbed directly or dissolved in air H_2_O aerosols, through stomata [62,63,64]. In the mesophyll, H_2_S is assimilated by *O*-acetyl-serine (thiol)lyase for the biosynthesis of cysteine [60]. The exogenous application of H_2_S has been described as a factor that increases tolerance to water deficit directly through a higher content of cysteine, and indirectly through the synthesis of metabolites such as proline and glycine betaine, and upregulating antioxidant enzymes [65].

Additionally, a synergistic interplay between nitric oxide (NO) and H_2_S signaling has been shown during stress events [66]. In anoxic soils, the aerenchyma of the root intervenes in the fixation of the H_2_S produced by microorganisms [67], so that the H_2_S of the soil can also trigger adaptive responses to stress in the radical tissues. On the other hand, the absorption and assimilation of SO_2_ and H_2_S in the leaves is a factor that modifies the nutritional status of the sulfur in the plant and decreases the activity of the SO_4_^2−^ transporters in the root [60].

In the case of COS, this gas is produced by biotic synthesis or by the oxidation of CS_2_ and DMDS. In plants, the SCN^−^ obtained from glucosinolate metabolism is hydrolyzed to COS and NH_3_. COS is the most stable and abundant gas form of S in the atmosphere (0.5 ppb). In the atmosphere, COS can be oxidized to sulfate to form aerosols, or be absorbed by plants and microorganisms that transform it into CO_2_ and H_2_S through carbonic anhydrase, RUBISCO, nitrogenase, and other metalloenzymes. In plants, COS can also be reduced to CS_2_, which is released back into the atmosphere [18]. The SO_4_^2−^ from the aerosols can be absorbed in the leaves by means of the co-transporter Sultr2;1 [62], located in the cells of the mesophyll.

### 3.2. Sulfur Assimilation and the Synthesis of H_2_S

**Figure 4:**
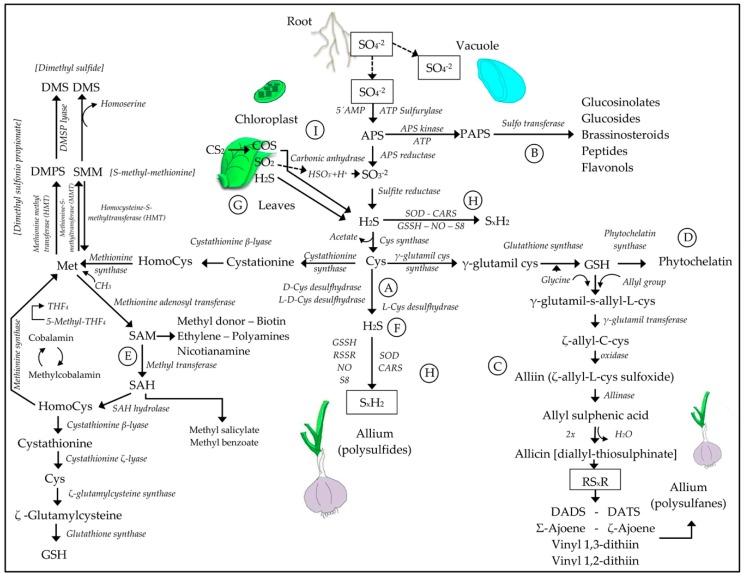
Schematic representation of the primary and secondary pathways of sulfur assimilation. In the primary assimilation pathway (**A**), APS is reduced to SO_3_ and subsequently to S^2−^/H_2_S, which are assimilated to form the amino acid cysteine [68,71]. In the secondary pathway (**B**), SO_4_ is phosphorylated and converted to 3′-phosphoadenosine 5′-phosphosulfate (PAPS) [48,68]. Cysteine is a central point for the synthesis of methionine or the production of polysulfanes (**C**), polysulfides (**H**), phytochelatins (**D**), SAM (**E**), and H_2_S (**F**) [48,68,72,73,74,75]. The absorption of sulfur in its gaseous forms is carried out by the stomatal route, directly incorporated into the primary pathway (SO_2_ and H_2_S) (**G**) [68], or through the action of carbonic anhydrase (COS) (**I**) [69,70].

Once SO_4_^2−^ is available in the cells from root absorption and transport, or by absorption of gaseous forms of S by the stomata, (Figure 4G,I) it is used to assimilate S into biomolecules [68,69,70]. The first step is to activate SO_4_^2−^ by means of the enzyme ATP sulfurylase. The resulting compound, adenosine 5′-phosphosulfate (APS), is used as a bifurcation between two assimilation pathways, primary and secondary. In the primary pathway, the APS is reduced by APS reductase to SO_3_^2−^, which in turn is reduced to S^2−^/H_2_S, which is assimilated into the amino acid cysteine (Figure 4A) [68,71]. Cysteine is the first product of the primary assimilation pathway of SO_4_^2−^, and is used for the synthesis of methionine and proteins, or as a donor of S^2−^ for the synthesis of a large number of metabolites, such as H_2_S (Figure 4F) [72], SAM, GSH, phytochelatins (Figure 4D,E) [48,68], SAM [68,73], polysulfides [72], and polysulfanes [74,75] (Figure 4C,H). In the secondary pathway (Figure 4B), SO_4_^2−^ is phosphorylated by APS kinase to 3′-phosphoadenosine 5′-phosphosulfate (PAPS) [48,68]. PAPS is the active sulfate donor for a variety of sulfation reactions in secondary metabolism. The reaction of sulfation is catalyzed by sulfotransferases, located mainly in the cytoplasm. To date, a large number of sulfated secondary compounds have been found to be involved in growth and stress signaling, and in the detoxification of environmental toxins [76,77].

As occurs in the metabolism of S in the soil (Figure 2), the great diversity of oxidation states of S allows the construction of a sophisticated and rich network of functional sulfur molecules for cell metabolism and signaling of plants. As in the soil, plants can exchange sulfur from the plant to the atmosphere and vice versa in the form of H_2_S, COS, and CS_2_, to incorporate the S in the assimilation pathways shown in Figure 4.

In recent years, both H_2_S and several of the derivative compounds (polysulfides and polysulfanes) or donors of this molecule have aroused great interest for their participation as oxidative stress reducers, in cellular signaling, and as post-translational modifiers. Because of its lipophilic nature, H_2_S is biologically reactive, since it can rapidly cross the membranes of cells without the intervention of channels. A possible H_2_S signaling mechanism is the formation of persulfides or hydrosulfides (RSSH) from the protein cysteine residues. It is assumed that H_2_S interacts in this way, with a great diversity of proteins such as channels, transcription factors, and enzymes [78,79].

H_2_S autooxidises in the presence of O_2_, forming polysulfanes, SO_3_^2−^, S_2_O_3_^2−^, and SO_4_^2−^ [70]; additionally, H_2_S is also a precursor of biological polysulfides [72]. Polysulfanes, polysulfides (with Sn > 2), and RSSH contain S^0^ atoms, which allows a diversity of oxidation states between the sulfur atoms and allows the molecules a dual character as oxidants and reducers. This diversity probably contributes to a multifunctionality character of the signaling of H_2_S and its derived compounds. Although H_2_S has a reactivity comparable to that of GSH against H_2_O_2_ and free radicals, it is believed that its value as a cellular antioxidant is limited because of its low concentration in vivo [78,79].

H_2_S donor compounds have been explored in the agricultural field for their possible applications in improving the productivity and quality of crops. It has been found that H_2_S mediates in signaling and in the increase in tolerance to different stresses such as heavy metals (Cd, Cr, Cu, Al, As), salinity, high temperature, and water deficit [17,65,80,81,82,83,84,85] Additionally, H_2_S and reactive sulfur species (RSS) interact with other relevant signaling molecules such as reactive oxygen (ROS) and reactive nitrogen (RNS) species [66,86], so the set of reactive chemical species could form a cellular network of redox signals [87]. These facts emphasize on the one hand the importance of adequate crop nutrition with S and, on the other hand, they highlight the advantages of the use of S^0^ applied to the soil and by dusting machine [33], because, presumably, S^0^ is a source of RSS as polysulfanes and polysulfides.

In addition to its relevance as a cell signaling and tolerance inducer, H_2_S is a source of RSS, a group of molecules of great biological importance that includes polysulfides and polysulfanes, SO_2_, S_2_O_3_^2−^, allicin, diallyl disulfide (DADS), and diallyl trisulfane (DATS), shown in Figure 4C,H. RSS can also be formed by the oxidation of thiols (e.g., GSH oxidized by H_2_O_2_). RSS are sulfur species capable of initiating oxidation reactions by nucleophilic substitutions, and can be non-radical or radical, as the thiyl radical RS*. RSS are not inactivated by antioxidants such as vitamins C and E or NADPH; for the above, GSH is required [88].

Polysulfides are inorganic RSS of the general formula RS_n_^2−^ (*n* > 2) such as H_2_S_2_^2−^, H_2_S_3_^2−^, H_2_S_4_^2−^, and H_2_S_5_^2−^. Polysulfides are produced metabolically by enzymatic catalysis, by partial oxidation of S^2−^ of H_2_S to produce H_2_S_n_, by reduction of H_2_S in the presence of polysulfanes, or by the reduction of polysulfanes in the presence of GSH. Polysulfides, depending on the molecule with which they interact, can behave as oxidants or reducers. It is considered that H_2_S_n_ polysulfides could be part of the signaling network and antioxidant impact currently attributed entirely to H_2_S [72]. The IUPAC [89] defines polysulfides as compounds R-[S]n-R, with a chain of S atoms *n* ≥ 2 and R ≠ H, however, in this manuscript we utilized the definition of Kharma et al. [72].

In soil, the abiotic synthesis of polysulfides occurs through the reaction between S^0^ and S^2−^, the oxidation of H_2_S by O_2_, H_2_O_2_, and possibly by iron oxides. Polysulfides are also produced by the bacterial oxidation of S^2−^ and constitute an essential substrate for both aerobic and anaerobic microbial metabolism, for example during S^0^ metabolism. In fact, it has been proposed that polysulfides and polysulfanes represent a critical part of the sulfur flux in ecosystems [44].

Polysulfanes are organic RSS with the general formula RSnH (R ≠ H, *n* ≥ 2). Polysulfanes contain S^2−^ and are very reactive with proteins and enzymes that contain cysteine; this characteristic possibly turns them into very versatile signaling agents [90].

The long-chain polysulfanes are characteristic molecules of the *Allium* species, with diallyl sulfanes in garlic and dipropyl sulfanes in onions [91,92]. The molecules frequently found in these species are allin (S-allyl-l-cysteine sulfoxide), diallyl trisulfane (DATS), diallyl tetrasulfane (DATTS), diallyl-pentasulfane (DAPS) or diallyl-hexasulfane, dimethyl-pentasulfane (DMPES), dipropyltrisulfane (DPTS), and dipropyl tetrasulfane (DPTTS), among others [90,91].

The compounds found in plants of the *Allium* genus have traditionally been used as plant protection products for both humans and crops [12]. This characteristic is believed to be related to the presence of polysulfides and polysulfanes in large quantities, 1.4% of fresh weight [93]. The reduced forms of polysulfanes can mitigate the impact of ROS such as superoxide and bind metal ions, decreasing oxidative stress in proteins and cell membranes [90]. The bioactivity of polysulfanes is enhanced by increasing the number of S atoms in the central functional groups of the molecule [94]. Pluth et al. [95] present in their review a large number of natural products that provide polysulfide-, polysulfane-, and H_2_S-releasing moieties.

The IUPAC [89] defines polysulfanes as having a chain > 2 of unbranched S atoms terminating in H:HSnH, however, this manuscript has utilized the definition of Kharma et al. [72].

## 4. Conclusions

The use of elemental sulfur (S^0^) as a sulfur source for plants has several advantages over the use of sulfate fertilizers. The long residence time in the soil, the activation of the soil microbiome, and the transformation of S^0^ into volatile sulfur species and reactive sulfur species that will induce higher tolerance to stress in plants were mentioned.

The process followed by sulfur was described, beginning with the application of S^0^ to the soil or foliage until it is transformed into sulfate, which is incorporated into the metabolism of sulfur in the plant through the primary and secondary pathways. Again, the application of S^0^ is associated with a great diversity of biomolecules that have a beneficial impact for plants, compared to the use of sulfur as sulfate.

The primary sulfate assimilation pathway was described as a process that gives rise to a great diversity of sulfur compounds, including H_2_S, polysulfides, and polysulfanes, which increase the nutritional quality of plants and increase tolerance to biotic and abiotic stresses.

The sulfur nutrition of plants, especially using S^0^ to cover all or part of the sulfur needs of both the soil and plants, should be explored with a higher intensity as a sustainable technique for the management and care of crops.

## Figures and Tables

**Table 1:** Representative sulfur compounds and their oxidation states.

Oxidation State	Representative Compound and Formula	Oxidation State	Representative Compound and Formula
+6	Sulfate, SO_4_^2−^	0	S^0^, elemental sulfur. Sulfoxide (R-S(-O)-R such as dimethyl sulfoxide (DMSO). Oxidized derivatives of sulfide and sulfenic acid (RSOH).
+6 and −2	Thiosulfate, S_2_O_3_^2−^	−1	Disulfide (R-S-S-R) is a persulfide found in the linkages between two cysteine residues in proteins. RSSH denotes persulfides (or hydrosulfides) obtained by the action of H_2_S on cysteine residues (R-SH). Thioethers and thiols can be oxidized to disulfides. Major products of decomposition of persulfides are polysulfanes. Thiyl-radical RS*.
+5 and −2	Polythionates (^−^O_3_S-S_n_-SO_3_^−^): Dithionate, S_2_O_6_^2−^; Trithionate, S_3_O_6_^2−^; Tetrathionate, S_4_O_6_^2−^	−2	Sulfide, S^2−^, polysulfides, S_2_^2−^, S_3_^2−^, S_5_^2−^; carbon disulfide (CS_2_); FeS_2_; NaHS and Na_2_S are sources of S^2−^ and of its conjugated acids SH^−^ and H_2_S. Polysulfides (with Sn > 2) contain S^0^ atoms, which allows a diversity of oxidation states.
+4	Sulfur dioxide, SO_2_; Sulfite, SO_3_^2−^; Disulfite, S_2_O_5_^2−^; Sulfone, OS(S) the oxidation product of sulfoxides	−2	Hydrogen sulfide (H_2_S), disulfane (H_2_S_2_), and polysulfanes (RSS_n_SR, *n* > 2). Polysulfanes contain S^0^ atoms, which allows a diversity of oxidation states.
+3	Dithionite, S_2_O_4_^2−^	−2	Thioethers (C-S-C) such as dimethyl sulfide (DMS), CH_3_-S-CH_3_ and dimethyl disulfide (DMDS), CH_3_-S-S-CH_3_.
+2	Carbonyl sulfide (COS), OCS	−2	Thiols (R-SH) such as glutathione (GSH) and methyl mercaptan, CH_3_-SH. Thiols are derived from the sulfhydryl group -SH of cysteine, which enables multiple oxidation states (−2 to +6). Thiolates are derivatives of thiols in which a metal or other cation replaces H.
0	Elementary sulfur (S^0^), mainly S_8_ (cycloocta-S)	−2	Carbon disulfide, CS_2_.
